# Psychometric Properties of the Subjective Cognitive Decline Questionnaire (SCD-Q) and Its Invariance across Age Groups

**DOI:** 10.3390/ijerph20021220

**Published:** 2023-01-10

**Authors:** Carmen Moret-Tatay, Iryna Zharova, Isabel Iborra-Marmolejo, Gloria Bernabé-Valero, María José Jorques-Infante, María José Beneyto-Arrojo

**Affiliations:** 1 MEB Laboratory, Faculty of Psychology, Universidad Católica de Valencia San Vicente Mártir, Avenida de la ilustración 2, 46100 Valencia, Spain; 2 Faculty of Psychology, National University of Ukraine on Physical Education and Sport, Kiev. Ukraine. St. Fizkul’tury, 1, 0315 Kyiv, Ukraine

**Keywords:** cognitive impairment, cognitive decline, ageing, SCD-Q

## Abstract

Considering that a good sense of subjective cognitive decline seems to be crucial to prevent decline before clinical impairment, the interest in examining tools on this front were raised in the last decade. The aim of the present study is to examine the psychometric properties of the Subjective Cognitive Decline Questionnaire (SCD-Q) across age in its Spanish adaptation. It should be noted that two constructs were proposed in this context: mnestic processes and executive function factors. For this reason, a sample of 750 individuals aged from 18 to 82 years participated in the study. They were divided into three different groups: young, middle, and older adults. A confirmatory factor analysis (CFA) and invariance analysis were carried out. Moreover, a logistic regression was employed to address the role of age. The results support a good goodness of fit for both uni- and bifactorial models. The invariance analysis reached the structural covariances levels. Last, age did not predict the recognition of cognitive decline in the last two years, while the SCD-Q bifactorial model did. These results are of interest both on a theoretical level, to provide more information on models of cognitive impairment, and on a practical level, for screening.

## 1. Introduction

Cognitive impairment is a widely recognized public health problem, with numbers expected to increase from 57.4 million cases in 2019 to 152.8 in 2050 [1]. The early detection of cognitive decline is a topic of interest to curb the nonreversible changes of dementia [2]. However, its detection is not as frequent as desirable [3,4,5]. Particularly, this study is of interest in the Spanish context. According to the latest data from the National Institute of Statistics, Spain has recently reached a new maximum ageing rate (133.5% in 2022).

During the first stages of cognitive impairment, subtle changes might occur, they being difficult to detect using objective tests [6]. Even if individuals are likely to experience cognitive changes throughout their lifespans, not all of them will consult a professional for advice [7]. In this context, a good sense of subjective cognitive decline (SCD) seems to be crucial as it might appear before clinical impairment [8], and it can be more sensitive than some neuropsychological tests [9,10]. Assessment tools such as the mini-mental state examination (MMSE) [11], a widely recognized screening tool with high sensitivity for detecting MCI and AD (Alzheimer’s disease), might not detect SCD or other symptoms in the first stages [8].

SCD can be defined as a self-perception of cognitive decline during an intermediate state between normal cognition and mild cognitive impairment (MCI) [12]. It should be noted that the presence of SCD is considered as a risk stage for Alzheimer’s disease (AD) and other dementias [13]. Thus, not surprisingly, research on this front has grown in the last decade [8]. However, SCD has received multiple names in the literature, such as subjective memory impairment (SMI), subjective cognitive complaint (SCC), and subjective memory complaint (SMC), among others [9,12,14]. As different cognitive symptoms have been defined in this context related to memory language, as well as to attention deficits, SCD seems to be the most appropriate approach [12,15].

One should bear in mind that SCD can be considered a very personal and introspective measure. Nevertheless, the literature has provided a large body of assessment tools in the field. One of the most popular ways of measuring this area is the use of questionnaires. After a systematic overview of the literature, authors such as Rabin et al. [16] identified 34 self-report measures in the field of SCD, comprising 640 cognitive self-report items. The same authors pointed out that psychometric strategies can be considered as essential tools to provide evidence-based procedures to select proper instruments in the characterization of SCD.

According to the COSMIC study on SCD across international cohort studies of aging, SCD is more frequent in old age [13]. However, to our knowledge, it is not clear how it is related to age in the literature, or when the primary onset of SCD first occurs. In addition, variables such as educational level may interfere in this context [13,17]. In this way, the aim of the present study was to examine the psychometric properties of the Subjective Cognitive Decline Questionnaire (SCD-Q) in its Spanish adaptation [18] that quantify the perceived subjective cognitive decline over the last two years. After a principal component analysis (PCA), the authors suggested a five-component structure. However, the percentage of explained variance was remarkably low for more than one factor (from 5.2% to 4.3%). Furthermore, the authors did not find any association between age or education and subjective decline measured in an older adult sample.

This study hypothesizes that the presence of SCD begins well before the age of 60 years. In this way, this approach was proposed in adults throughout their lifespans to study the effect of age, also considering the education variable. Focusing on the tool under study [18], it is possible to identify content for two constructs in its items: mnestic processes (items 3–15 and 17) and executive functions (items 1, 2, 16 and 18–24). Although the authors’ initial proposal is one-factor, a two-factor solution is hypothesized because of the item content. Last, considering that the beginning of the onset of cognitive impairment can start at any age, invariance is hypothesized with other groups of adults, other than older adults.

## 2. Materials and Methods

### 2.1. Participants

The initial sample comprised 750 individuals aged from 18 to 82 years old, controlling the sex variable for a balanced sample of men and women (as explained for each age groups). To be a Spanish adult (participants had to be over 18 years old) and unimpaired functional ability were considered inclusion criteria, while the exclusion criteria involved the exclusion of major depression and anxiety disorders reported by participants. Participants from 49 different communities in Spain took part in the study. No differences were found on the SCD-Q final score after performing the Kruskal–Wallis test across place of origin.

A total of 58.9% of the participants were actively working, 10% were unemployed, 23% were students, 20.8% were retired, and 6.3% were housekeepers. In relation to marital status, 58.5% were married, 9.6% in a romantic couple, 9.2% separated or divorced, 19.7% single, and 2.9% widowed. A total of 34.5% reported worse cognition in the last two years.

The participants were divided into three groups: young, middle, and older adults. The young adults (*n* = 251) ranged from 18 to 40 years old (with an age M_ean_ = 32.34, SD = 5.72, and 49.8% of women), the middle adults (*n* = 250) ranged from 41 to 60 years old (with an M_ean_ = 49.97, SD = 5.45 and 52.4% of women), and the older adults (*n* = 249) ranged from 61 to 82 years old (with an age M_ean_ = 66.68, SD = 4.81 and 49% of women).

With regards to education, in the younger group, 12.35% of the participants reported to have only completed primary school, 26.3% specialization studies, and 61.36% higher or further education. In the middle group, 19.6% of the participants reported to have only completed primary school, 34% specialization studies, and 46.4% higher or further education. Last, in the older group, 18.9% have only completed primary school, 33.7% specialization studies, and 47.4% higher or further education.

### 2.2. Materials and Procedure

All participants answered a sociodemographic list of questions, as well as the validated SCD-Q questionnaire in the Spanish population [18]. The questionnaire involves 24 items administered in parallel to the target participants (Part I also named MyCog) and to their informants (part II also named TheirCog). In this case, only the participants version, MyCog, was employed, as TheirCog was considered as a convergent measure. A Cronbach alpha score of 0.90 was described by the authors for the whole dataset. The specific SCD-Q questions can be found in the original paper by Rami et al. (2014). Moreover, the question, “In the last two years, has your cognition or memory declined? (Yes/No)” has been included, as in the original study, for a logistic regression analysis.

The sample recruitment was incidental, a non-probabilistic procedure using a snowball approach. Participants completed the questionnaire in a self-administered online form. The ethics committee approved the study (UCV/2020-2021/163), and all participants signed informed consent to participate in the study.

### 2.3. Analysis

The first part of the study focused on factor analysis with a sample of older people, followed by a multi-group analysis with other age groups. Last, the relationship between age and differences according to education was studied.

It should be noted that the 24-item SCD-Q Spanish version employs tetrachoric estimators under dichotomous measured variables. A factor analysis was conducted using the robust unweighted least squares (RULS) method of extraction. Parallel analysis [19] for the exploratory method was employed based on minimum rank factor analysis [20]. An estimation for tetrachoric correlation matrix was employed to reveal the factor structure of the older participant’s subsample. A Promax option was chosen since the factors correlated and an overlap across them is suggested [21]. For the confirmatory factor analysis (CFA), the goodness of fit was assessed through the chi-square [22], the comparative fit index (CFI) whose values range from between 0 and 1 and the reference value is 0.90 [23] and, for the parsimony adjustment indices, the error of the root mean square approximation (RMSEA) where the smaller the value, the better the fit, the reference value being 0.05 [24].

For the analysis of invariance, a hierarchical procedure was carried out following different levels of restriction: beginning with an unconstrained model and adding constraints successively. The logic of this procedure is to test the factorial homogeneity structure across groups, from a stage where all parameters do not need to be equal to a stage where they must be. In this way, several authors [16,25] recommend the invariance analysis on the development of a psychometric test, particularly when comparing different populations [26]. As in CFA, the goodness of fit was assessed through chi-square, CFI, and RMSEA. The analyses were run using the FACTOR software v12.03.02 [27] and Amos v18 (IBM).

## 3. Results

First, an exploratory analysis was carried out on the older sample. In this way, Table 1 depicts univariate descriptive analyses in the older sample for the SCD-Q (*n* = 249). As the correlation matrix was not positive definite, being some of the eigenvalues related to the correlation matrix negative (number of negative eigenvalues = 3), a sweet smoothing algorithm was employed [28], as well as the PCDi (percentage of covariance destroyed in each variable). PCDi ranged from 40.7 to 10.7% and was considered fair (22%). Table 2 depicts the tetrachoric correlation in the older sample for the SCD-Q. In terms of scale reliability statistics, both Cronbach’s α and McDonald’s ω for the 24-item solution were =0.936.

The results of the Kaiser–Meyer–Olkin measure of sampling adequacy (KMO = 0.93, bootstrap 95%CI = 0.90−0.96) and the Bartlett’s test of sphericity (BTS = 2753.5, df = 276 *p <* 0.001) indicated that the data were suitable for factor analysis. The factor loadings of the 24 items under one- and two-factor solutions are also shown in Table 1. An oblique rotation was chosen for the two-factor solution as this procedure assume correlation among factors. Other solutions for three and four dimensions were not considered, as the explained variance was lower than 5% and there were no loadings lower than the absolute value of 0.30 in one of the factors. The parallel analysis suggested four factors as the eigenvalue for a five-factor solution was =0.92, while for a four-factor solution it was =1.05. The proportion of explained variance for factor one was =41.5%, but for factors 2 to number 4 they ranged from 5 to 4%. Cronbach’s α and McDonald’s ω for the 24-item solution were =0.936.

For the confirmatory factor analysis, item numbers 16 and 19 were not considered, as they did not discriminate between F1 and F2 and showed lower factor loading. The chi-square test was significant, indicating an optimal fit for one factor solution (χ^2^ = 406.453, df = 252, *p* < 0.001). The comparative fit index (CFI = 0.92) and the root mean square error approximation (RMSEA = 0.056; 95%CI = 0.052–0.060) were optimal. With regards to the two-factor solution, the factor loadings followed the proposed model, except for items 16 and 19. These items did not discriminate between factors 1 and 2. In this case, and following a holistic criterion, they were considered in the factor of theoretical origin, executive functions. The chi-square test was significant, indicating an optimal fit for a one-factor solution (χ^2^ = 363.776, df = 229, *p* < 0.001). The comparative fit index (CFI = 0.99) and the root mean square error approximation (RMSEA = 0.049; 95%CI = 0.042–0.053) were optimal. Cronbach’s α and McDonald’s ω for the F1 were α = 0.880 and ω = 0.884, as well as α = 0.893 and ω = 0.896 for the F2, respectively.

Second, an analysis of invariance was carried out across young (*n* = 251), middle (*n* = 250), and older adults (*n* = 249). Differences can be noticed in the increases (Δ) in the indices under study. Table 3 depicts four levels of restriction: i) Model 1 = unconstrained; Model 2 = measurement weights; Model 3 = structural covariances; and Model 4 = measurement residuals. According to Table 3, there are no significant changes comparing Model 4 with the less-constrained than with the previous Model 3 or baseline. Two other upper models could be added that were not considered as they did not accept Model 4: saturated and independence models.

As expected, F1 and F2 were strongly correlated: r = 0.80; *p* < 0.01. A negative correlation was found between the variable age and F1 (executive functions) conditioned on the variable education: r = −0.09; *p* < 0.05. No relationship was found across age and F2 (memory). Moreover, through an analysis of variance (ANOVA), no differences between education groups were found across F1 and F2 scores (all *p* > 0.05). Last, a logistic regression was carried out where age and SCD-Q factors (F1 and F2) were entered as predictors. The outcome was the answer to the question: in the last two years, has your cognition or memory declined? (*Yes/No*). The model suggests a statistically significant relationship between outcome and predictor variables F1 and F2, but not age: χ^2^_(746)_ = 286.787; *p <* 0.01; McFadden R^2^ = 0.297. As shown in Table 4, the odds ratio value shows the power of F1 in predicting the outcome, as well as aging being not statistically significant.

**Figure 1:**
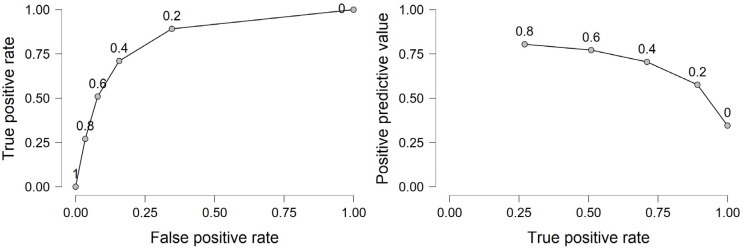
Sensitivity and specificity for the logistic model under study.

As shown in Figure 1, the sensitivity was 0.622 and specificity = 0.882. The receiver operating characteristic curve (AUC) was =0.857.

## 4. Discussion and Conclusions

The aim of this work was to study the SCD-Q questionnaire in its Spanish adaptation. In this case, three age groups were included, not only older adults as commonly done in the previous literature. Psychometric properties, factor structure, and its relationship with age were analyzed, including an analysis of invariance.

As the previous literature suggests [16], the review of psychometric properties is of interest to optimize measurement instruments, e.g., by reducing items if necessary. In this case, we propose to review the structure of the SCD-Q questionnaire and the reduction of two items (numbers 16 and 19). While the authors who proposed the Spanish tool [18] have opted for a single-factor structure based on statistical criteria, a two-factor structure is proposed in the current study. This attempt is based on a holistic approach to content. More precisely, these two factors can be described as memory processes (e.g., remembering familiar events or recent conversations), as well as executive functions (e.g., concentration or planning). Bearing in mind previous literature in the field, one should bear in mind that memory is one of the main problems reported by individuals in case of cognitive impairment [29], while executive functions are among the first symptoms related to vulnerable anatomical areas in the brain associated with age. The present results would support both unifactorial and bifactorial structure. However, the two-factor solution was chosen because of the possibility of individualizing the two cognitive processes of interest in general terms. Nevertheless, it is not surprising that a unifactorial structure can be proposed and be very useful, as memory and executive functions are highly related in the literature [30,31].

In relation to the invariance analysis, the age groups achieved a high level of constraint (structural covariances). This would support the use of the questionnaire in different age groups and specifically in the proposed two-factor version. In addition, the logistic analysis showed that age has no effect on the subjective impairment reported by the participants in the last two years. This result supports that subjective decline may occur at any age, as stipulated by the previous literature [32]. According to Taylor, Bouldin, and McGuire [33], SCD is increased with age. More precisely, the authors reported a prevalence of 10.4% among adults aged 45–54 years to 14.3% among those aged ≥75 years. Current results support this trend for executive functions, more than memory losses. The same authors also reported that SCD was lower among college graduates (7.0%) than among individuals who only attended high school education (18.2%). However, current results did not support this difference. One reason could be the way of classifying participants into only three levels of education. Future studies should address not only the qualifications achieved but also the years of study.

The innovation of this study is to consider a new structure of the factors under study. According to the literature in the field, subjective memory is of interest to implement objective measures of memory abilities [34], and revisiting factor structure might improve evidence in this front. However, a concept of memory and executive functions has been addressed in general terms and should be specified for future studies (e.g., prospective memory). This study has several limitations. First, groups with a cognitive impairment level were not included for the purpose of studying the discrimination of the questionnaire. Second, this is a self-administrated questionnaire under a cross-sectional design. Thus, some bias might occur. Last, although participants were asked to indicate any medical conditions of concern, neurological or psychiatric alterations were not addressed in a specific way—a subject of interest for subjective complaints.

The current results allow us to conclude good goodness of fit for both uni- and bifactorial models, as well as invariance up to the structural covariances level. Age did not predict the recognition of cognitive decline in the last two years, while the SCD-Q bifactorial model did. These results are of interest at both theoretical and applied levels, first, to clarify the theoretical definition of SCD models underlying assessment tools, and second, to implement SCD screening, and ultimately, to promote earlier treatment if necessary.

## Figures and Tables

**Table 1:** Univariate descriptives and factor loadings in the older sample for the SCD-Q (*n* = 249). Bold indicates the factor loading items included for a 2-factor solution (values > 0.40).

		CI 95% Levels					One-Solution	Two-Solutions	
Variable	Mean	Lower	Upper	Variance	Skewness	Kurtosis	F1	F1	F2
Item 1	0.414	0.33	0.49	0.24	0.243	0.352	0.577	0.122	**0.654**
Item 2	0.249	0.18	0.32	0.18	0.187	1.166	0.687	0.130	**0.586**
Item 3	0.297	0.22	0.37	0.22	0.209	0.891	0.644	0.356	**0.478**
Item 4	0.233	0.16	0.30	0.16	0.179	1.269	0.667	**0.588**	0.274
Item 5	0.233	0.16	0.30	0.16	0.179	1.269	0.710	0.369	**0.529**
Item 6	0.165	0.10	0.22	0.10	0.138	1.816	0.654	**0.537**	0.174
Item 7	0.317	0.24	0.39	0.24	0.217	0.788	0.520	**0.865**	−0.162
Item 8	0.161	0.10	0.22	0.10	0.135	1.856	0.546	**0.746**	−0.127
Item 9	0.249	0.18	0.32	0.18	0.187	1.166	0.690	**0.789**	0.098
Item 10	0.386	0.31	0.46	0.31	0.237	0.472	0.631	0.129	**0.704**
Item 11	0.221	0.15	0.29	0.15	0.172	1.351	0.747	**0.888**	0.060
Item 12	0.522	0.44	0.60	0.44	0.250	−0.089	0.533	**0.662**	−0.041
Item 13	0.333	0.26	0.41	0.26	0.222	0.710	0.607	**0.526**	0.271
Item 14	0.317	0.24	0.39	0.24	0.217	0.788	0.687	**0.501**	0.382
Item 15	0.349	0.27	0.43	0.27	0.227	0.634	0.550	0.301	**0.431**
Item 16	0.177	0.11	0.24	0.11	0.145	1.702	0.579	0.325	0.314
Item 17	0.221	0.15	0.29	0.15	0.172	1.351	0.683	**0.803**	0.079
Item 18	0.257	0.19	0.33	0.19	0.191	1.116	0.628	−0.018	**0.840**
Item 19	0.153	0.09	0.21	0.09	0.129	1.940	0.641	0.396	0.311
Item 20	0.137	0.08	0.19	0.08	0.118	2.126	0.473	0.013	**0.548**
Item 21	0.329	0.25	0.41	0.25	0.221	0.729	0.604	−0.080	**0.878**
Item 22	0.120	0.07	0.17	0.07	0.106	2.341	0.676	0.075	**0.705**
Item 23	0.269	0.20	0.34	0.20	0.197	1.046	0.636	0.161	**0.675**
Item 24	0.249	0.18	0.32	0.18	0.187	1.166	0.547	−0.039	**0.774**

**Table 2:** Tetrachoric correlation in the older sample for the SCD-Q (*n* = 249).

*	I1	I2	I3	I4	I5	I6	I7	I8	I9	I10	I11	I12	I13	I14	I15	I16	I17	I18	I19	I20	I21	I22	I23	I24
I1	1																							
I2	0.56	1																						
I3	0.54	0.52	1																					
I4	0.57	0.65	0.58	1																				
I5	0.57	0.66	0.76	0.67	1																			
I6	0.55	0.62	0.57	0.67	0.64	1																		
I7	0.55	0.52	0.52	0.58	0.50	0.48	1																	
I8	0.57	0.64	0.44	0.73	0.54	0.69	0.62	1																
I9	0.64	0.60	0.62	0.71	0.66	0.70	0.61	0.71	1															
I10	0.72	0.61	0.53	0.62	0.59	0.57	0.55	0.62	0.65	1														
I11	0.58	0.60	0.67	0.69	0.60	0.72	0.70	0.70	0.73	0.57	1													
I12	0.54	0.53	0.55	0.54	0.48	0.52	0.62	0.55	0.55	0.62	0.65	1												
I13	0.57	0.58	0.52	0.61	0.53	0.63	0.53	0.62	0.63	0.56	0.66	0.64	1											
I14	0.64	0.66	0.57	0.65	0.58	0.56	0.57	0.60	0.59	0.63	0.62	0.67	0.71	1										
I15	0.58	0.64	0.58	0.58	0.56	0.55	0.51	0.51	0.60	0.65	0.59	0.61	0.58	0.59	1									
I16	0.55	0.62	0.52	0.69	0.62	0.61	0.59	0.59	0.62	0.62	0.66	0.53	0.52	0.58	0.54	1								
I17	0.63	0.63	0.60	0.70	0.66	0.64	0.61	0.67	0.67	0.55	0.74	0.69	0.68	0.77	0.61	0.63	1							
I18	0.59	0.65	0.57	0.64	0.64	0.63	0.50	0.60	0.73	0.65	0.62	0.53	0.61	0.58	0.63	0.62	0.59	1						
I19	0.55	0.57	0.60	0.67	0.63	0.63	0.58	0.61	0.64	0.60	0.70	0.43	0.51	0.60	0.52	0.67	0.66	0.66	1					
I20	0.55	0.56	0.53	0.56	0.56	0.59	0.43	0.57	0.56	0.58	0.61	0.34	0.48	0.48	0.52	0.63	0.53	0.61	0.74	1				
I21	0.56	0.54	0.52	0.52	0.55	0.57	0.55	0.47	0.57	0.56	0.55	0.48	0.51	0.53	0.58	0.51	0.55	0.67	0.58	0.59	1			
I22	0.56	0.65	0.55	0.66	0.58	0.62	0.55	0.62	0.63	0.60	0.59	0.45	0.55	0.58	0.56	0.72	0.58	0.72	0.64	0.60	0.71	1		
I23	0.62	0.57	0.52	0.52	0.53	0.53	0.49	0.57	0.59	0.58	0.58	0.40	0.52	0.59	0.56	0.52	0.52	0.60	0.49	0.53	0.61	0.65	1	
I24	0.58	0.59	0.56	0.59	0.55	0.58	0.53	0.59	0.61	0.67	0.62	0.52	0.53	0.58	0.52	0.64	0.55	0.68	0.66	0.62	0.73	0.70	0.59	1

* I = item from the SCD-Q item.

**Table 3:** Goodness-of-fit statistics for tests of invariance across young (*n* = 251), middle (*n* = 250), and older adults (*n* = 249).

Model *	χ^2^	df	χ^2^/df	CFI	RMSEA	Δ χ^2^	Δ df	Decision
Model 1	12550.19	624	20.01	0.908	0.037	-	-	-
Model 2	13020.79	664	10.96	0.907	0.036	470.6	40	Accept
Model 3	13130.96	670	10.96	0.906	0.036	110.17	6	Accept
Model 4	15390.54	714	20.15	0.880 *	0.039	2259.58 *	44	Reject

* Model 1 = unconstrained; Model 2 = measurement weights; Model 3 = structural covariances; Model 4 = measurement residuals.

**Table 4:** Logistic regression coefficients.

					Wald Test			95% Confidence Interval	
	Estimate	Standard Error	Odds Ratio	z	Wald Statistic	df	*p*	Lower Bound	Upper Bound
(Intercept)	−2.013	0.357	0.134	−5.644	31.859	1	<0.001	−2.712	−1.314
Age	−0.009	0.006	0.991	−1.430	2.046	1	0.153	−0.022	0.003
F1 *	3.466	0.487	31.997	7.115	50.626	1	<0.001	2.511	4.420
F2 *	1.848	0.456	6.350	4.050	16.399	1	<0.001	0.954	2.743

* F1 (executive functions); F2 (memory).

## Data Availability

The data presented in this study are available on request from the corresponding author.
